# Context-dependent similarity effects in letter recognition

**DOI:** 10.3758/s13423-015-0826-3

**Published:** 2015-04-09

**Authors:** Sachiko Kinoshita, Serje Robidoux, Daniel Guilbert, Dennis Norris

**Affiliations:** ARC Centre of Excellence in Cognition and its Disorders (CCD), Department of Psychology, Macquarie University, Sydney, NSW 2109 Australia; ARC Centre of Excellence in Cognition and its Disorders (CCD), Department of Cognitive Science, Macquarie University, Sydney, NSW Australia; Department of Psychology, Macquarie University, Sydney, NSW Australia; MRC Cognition and Brain Sciences Unit, Cambridge, UK

**Keywords:** Letter identification, Orthographic processing, Abstract letter identity

## Abstract

In visual word recognition tasks, digit primes that are visually similar to letter string targets (e.g., 4/A, 8/B) are known to facilitate letter identification relative to visually dissimilar digits (e.g., 6/A, 7/B); in contrast, with letter primes, visual similarity effects have been elusive. In the present study we show that the visual similarity effect with letter primes can be made to come and go, depending on whether it is necessary to discriminate between visually similar letters. The results support a Bayesian view which regards letter recognition not as a passive activation process driven by the fixed stimulus properties, but as a dynamic evidence accumulation process for a decision that is guided by the task context.

**Electronic supplementary material:**

The online version of this article (doi:10.3758/s13423-015-0826-3) contains supplementary material, which is available to authorized users.

To read a word, the letters comprising the word must be identified. There is a general consensus that the word recognition process involves abstract letter identities, which allows skilled readers to recognize letters across variations in allography (e.g., g and G) and font (e.g., g and g). Beyond this general consensus however, the letter recognition process in current visual word recognition models is not very well specified (Finkbeiner & Coltheart, 2009). The present study investigates the role of visual similarity in letter recognition to shed light on this important “front-end” of reading.

Visual similarity has a puzzling effect on letter recognition in masked priming. On the one hand, it has a robust effect when the prime is not a letter, as evidenced by the “leet priming” phenomenon. Leet primes contain digits or other symbols that resemble the letters in a target word (e.g., M4T3R14L, M∆T€R14L): These primes facilitate the recognition of the target word (MATERIAL), much more than primes that contain visually dissimilar digits/symbols (e.g., M5T6R28L; Perea, Dunabeitia, & Carreiras, 2008). In contrast, few studies have reported a parallel effect with *letter* primes, reporting that visually similar letter primes produce no more priming than visually dissimilar letter primes (e.g., the letter H, which is visually more similar to the letter A, produces no more priming than the letter D). Recently, using the same target words, Kinoshita, Robidoux, Mills, and Norris (2014) reported a clear dissociation in the effect of visual similarity between digit and letter primes.

In Kinoshita et al.’s (2014) masked priming lexical decision study, the primes were generated by substituting letters in the word target (e.g., the letters A, and B in ABANDON) with visually similar digits (e.g., 484NDON), visually similar letters (e.g., HRHNDON), visually dissimilar digits (e.g., 676NDON), and visually dissimilar letters (e.g., DWDNDON). Critically, the substituted letters and digits were matched on confusability with the target letter. Despite being matched on the degree of visual similarity, the amount of priming effects produced by digit (leet) primes was dependent on visual similarity, but the amount of priming produced by letter primes was not.

Kinoshita et al. argued that this puzzling dissociation is explained naturally by a Bayesian view of word recognition (e.g., the Bayesian Reader: Norris, 2006; Norris & Kinoshita, 2012). According to this view, letter recognition is not a passive process in which letter representations are “activated” by the perceptual input, but rather a dynamic process of accumulating evidence for a decision. Primes similar to the target produce priming not because they activate a perceptually similar target, but because they contribute evidence for the target over alternatives in the decision-making process. In the lexical decision task, the targets are strings of letters and the goal of the task is to decide whether the letter string corresponds to a word. In this context, the input contains only letters, and never digits. Since there is no need to distinguish digits from letters, a digit (e.g., 4) can contribute evidence for the presence of a visually similar letter (e.g., A). In contrast, all of the *letters* must be distinguished from each other so that the task requires that each letter (e.g., H) contributes evidence only for itself, and not for other letters (e.g., A). That is, whether the visual similarity between two symbols (A and H; A and 4) produces priming is not determined solely by the degree of physical similarity between them, but is modulated by the nature of evidence required to make the decision – here, by whether the symbol H or 4 is contained in the set of target symbols from which the symbol A needs to be distinguished.

The Bayesian account is not the only explanation for the dissociation between digit and letter primes, however. An alternative would be to argue that the dissociation reflects different properties of digit and letter representations. For example, within the interactive-activation framework adopted in many models of visual word recognition (e.g., the Dual Route Cascaded model, Coltheart, Rastle, Perry, Langdon, & Ziegler, 2001; the Spatial Coding model, Davis, 2010), this could be implemented as lateral inhibitory connections between representations. To accommodate the dissociation between digit and letter primes, the inhibitory connections between letters may be set to be stronger than those between letters and digits.

The present study tests these accounts. If the dissociation between the digit and letter primes reflects a fixed property of letter/digit representations as in activation-based accounts, then the visual similarity effect with letter primes should always be smaller than with digit primes. In contrast, if the letter recognition process is an active perceptual decision-making process then it should be possible to make the effect of letter similarity come and go as a function of the decision required. To this end, we turn to the same-different task which allows us to construct sets of targets that differ in terms of which letters need to be discriminated from each other. In this task, participants are first presented with a referent, and are asked to decide whether a subsequently presented target is the same as, or different from, the referent. The masked priming procedure is the same as in a lexical decision task: Following a forward mask consisting of #’s, a prime is presented briefly, and is in turn backward-masked by the target so that participants are unaware of the identity of the prime.

The standard account of masked priming is that the prime “‘activates” the target. According to this view, priming should be a simple function of the relation between the prime and the target. However, contrary to this view, Norris and Kinoshita (2008) showed that in the same-different task, as predicted by the Bayesian Reader, masked priming effects are found with the “Same” trials (where the target is the same as the referent) but not with the “Different” trials (where the target is different from the referent). This prediction was based on two assumptions. The first is that the prime and target are treated as a single perceptual event. The second is that perception involves a gradual accumulation of evidence contributing to the decision required to perform the task. Masked priming is a consequence of the way evidence accumulated from the prime contributes towards the decision required to the target. In a “Different” trial, e.g., when the referent is “fist” and the target is “PAGE”, the identity prime “page” provides evidence towards the decision that it is different from the referent. However, an unrelated control prime “ship” would also provide evidence that it is different from the referent. The net result is no difference in the amount of priming effect produced by the two types of prime. (The same logic explains why in the lexical decision task masked priming effects are limited to the “Word” trials – see Norris & Kinoshita, 2008, for detail.) Other accounts of masked priming (e.g., Bowers, 2010) attempt to explain this pattern in terms of a mixture of lexical priming and response bias. They assume that a masked prime will be partially identified such that a prime that is different to the referent will bias the decision towards “Different,” while a prime that is the same will bias the decision towards “Same,” counteracting the priming benefit due to the shared identity between the prime and the target. However, the absence of priming in the “Different” trials cannot be explained by a bias to respond “Different” offsetting the priming benefit due to the shared identity between the prime and the target. Additionally, there is now considerable empirical evidence against this view (Kinoshita & Norris, 2010, 2011; Norris & Kinoshita, 2010).

In the present same-different task, the referents (and the “Same” target strings) always consisted of three-letter strings comprised of the letters A, B, I, and S (the critical letters used in Kinoshita et al., 2014), e.g., ABI. As in Kinoshita et al., the critical primes were formed by replacing each letter in the referent with either a visually similar or dissimilar digit, or a visually similar or dissimilar letter. For the referent/target ABI, for example, a similar digit prime (e.g., 481) would therefore be a form of leet prime; a visually similar letter prime would be HRL; a visually dissimilar digit prime would be 673, and a visually dissimilar letter prime would be DWG. To test the activation-based account against the Bayesian Reader account, we manipulated the nature of the Different targets. For one group of subjects, the Different targets were constructed from the same set of letters as the similar primes (HRL; the Overlapping Target Context). That is, the letters that made up the primes also appeared in the target strings, much as they would in a lexical decision task. In this context, each prime letter (e.g., H) has to be distinguished from other letters including the visually similar referent letter (e.g., A) – that is, the letter H cannot be taken as an instance of the letter A. In this condition, both accounts predict the similarity effect for the letter primes to be absent. In the other condition (the Distinct Target Context), the Different target strings were constructed from a set of letters that did not appear in any primes or referents (e.g., CZE). This changes the nature of the evidence required to make the same-different decision, as it is no longer necessary to discriminate the visually similar letter H from the referent/target letter A. In this context, the Bayesian Reader account would predict a similarity effect to emerge for the letter primes because, here, the visually similarity of the prime letter H to A can contribute evidence for the letter A, while any accounts that assume fixed processing across contexts will continue to predict no visual similarity effect for letters. For digit primes, both accounts predict that the primes will replicate the visual similarity effects that have been observed in previous studies. This is expected irrespective of context, because digits never appear as targets or referents in either context.

## Experiment

### Method

#### Participants

Forty-seven students from Macquarie University, Sydney, NSW, Australia, participated in this study for course credit. Twenty-two were assigned to the Overlapping Target Context and 25 were assigned to the Distinct Target Context, in the order of arrival.

#### Design

The experiments used the masked priming same-different task involving three-letter strings (e.g., ABI). The critical manipulations concerned the nature of primes in the “Same” condition (where the referent and the target strings were the same), crossed with the nature of “Different” targets (Target context). There were four critical within-participant prime conditions resulting from a factorial manipulation of Prime type (Letter or Digit) and Similarity (Similar, Dissimilar). In addition to the four critical conditions, we included an Identity prime condition as a check on our manipulation. Target Context was a between-participant manipulation. In the Overlapping Target Context, the targets on “Different” trials were comprised of the Similar prime letters; in the Distinct Target Context, they were comprised of letters that did not overlap with the Similar or Dissimilar letter primes (see Materials). The dependent variables were response latency and error rate.

#### Materials

The critical stimuli were 24 letter-string targets, with each item consisting of three uppercase letters with no repetitions. Each target contained the letters A, I, S, or B. These letters were chosen because they had digits that resembled them (A/4, I/1, S/5, and B/8), which were used to construct the Similar Digit primes (e.g., 481 for the target ABI). In addition to the visually similar digits, for each of the letters, we chose a visually dissimilar digit (A/6, I/3, S/9, and B/7), a visually similar letter (A/H, I/L, S/E, and B/R), and a visually dissimilar letter (A/D, I/G, S/M, and B/W). These letters were chosen from a wider range of items selected by consulting Mueller and Weidermann’s (2012) letter confusion matrices, which were then tested in a 2AFC identification task to ensure the digits and letters were equated on their degrees of similarity to the base letter (see Kinoshita et al., 2014).

For each target string, five primes were generated. The four critical prime conditions were a factorial combination of the factors Similarity (Similar vs. Dissimilar) and Prime Type (Letter vs. Digit). In the Similar-Letter condition, the prime was constructed by substituting each letter in the target with its associated similar letter (e.g., HRL–ABI). In the Dissimilar-Letter condition, the prime was constructed by substituting the associated dissimilar letters (e.g., DWG–ABI). The same procedure was used to generate the Similar- (e.g., 481–ABI), and Dissimilar-Digit primes (e.g., 673–ABI). The fifth, Identity, prime (e.g., ABI-ABI) condition was included as a manipulation check and will not be discussed in the analysis.

On the “Same” trials, each target was simply the referent presented 1.2 times larger (e.g., referent – ABI, target-ABI). On the “Different” trials, the targets consisted of three letters that were different from the referent. In the Overlapping Target Context, the “Different” targets consisted of the letters that appeared in the Similar letter primes (e.g., referent – ABI, target-HRL); in the Distinct Target Context, the “Different” targets consisted of the letters C, Z, E, and Y, which were not used in any primes or referents (e.g., referent – ABI, target-CZE).

The entire set of stimuli is listed in the Appendix.

#### Apparatus and procedure

Participants were tested individually or in pairs, seated approximately 60 cm away from the stimuli presented on a flat-screen monitor. Each participant completed 240 test trials consisting of 120 “Same” and 120 “Different” trials, presented in two half blocks with each half block containing an equal number of “Same” and “Different” trials, and an equal number of trials from each prime conditions. There was a self-paced break between the blocks. The trial order was randomly generated for each participant.

Participants were instructed that on each trial they would be presented with a referent, followed by a target, and that their task was to decide whether the target was the same as or different from the referent, as quickly and accurately as possible. No mention was made of the prime. They were instructed to press a key marked “+” on a response pad for “Same” response and a key marked “−” for a “Different” response.

Stimulus presentation and data collection were controlled by the DMDX experimental software (Forster & Forster, 2003). Stimulus display was synchronized to the screen refresh rate (10.01 ms).

Each trial started with the presentation of a referent above a forward mask consisting of three hash marks (###) in 10-point Courier New Font for 1 s. The referent disappeared, and the forward mask was replaced by the prime presented in uppercase letters in 10-point Courier New Font for 40 ms, which in turn was replaced by the target presented in uppercase letters in 12-point Courier New Font until the participant’s response. The prime and the target were presented in different-sized fonts to avoid physical continuity. Participants were given feedback (“Wrong response” message on the screen) only when they made an error on the trial.

### Results

The mean reaction times (RTs) for the correct trials and the error rates for the five prime conditions in the two contexts (differing in the nature of the “Different” targets used) for the “Same” trials are shown in Table 1.

**Table 1:** Mean response latencies (reaction times, RTs, in ms) and percent error rates (%E) for the SAME responses in Experiment 1 (same–different match task)

		Distinct target context		Overlapping target context	
Prime type	Example	RT	%E	RT	%E
Referent – ABI; Target – ABI					
Identity	ABI	409	1.7	402	1.3
Similar letter	HRL	465	6.7	487	10.6
Dissimilar letter	DWG	495	6.5	485	7.2
Letter similarity effect		30	−0.2	−2	−3.4
Similar digit	481	435	3.2	421	2.8
Dissimilar digit	673	476	6.8	469	5.5
Digit similarity effect		41	3.6	47	2.7

The mean RTs for the “Different” trials were 472 ms in the Distinct target context and 464 ms in the Overlapping target context

The RT data were analyzed using linear mixed effects modeling (Baayen, 2008). The preliminary treatment of RT data for this analysis was as follows. First, we examined the shape of RT distribution for correct trials requiring the SAME response (a total of 5346 observations), and applied an inverse transformation (1/RT) to best approximate a normal distribution, in order to meet the distributional assumption of the linear mixed effects model. We excluded trials with RTs shorter than 200 ms (1 data point) as an outlier.

The data were submitted to a linear mixed–effects model using the lme4 (Bates, Maechler, Bolker, & Walker, 2013, version 1.1-5) package implemented in R 3.0.3 (R Development Core Team, 2014-03-06). As it was of no theoretical interest, we excluded data from the identity prime condition. The model included as fixed factors the RTs on the previous trial (PrevRT), along with a full factorial combination of Prime type, Similarity, and Target Context, which were all deviation-contrast-coded (−.5, .5). Intercepts for subjects and items, and by-subject and by-item random slopes for the effects of Similarity were included as crossed random factors, in line with the recommendation to keep the random effect structure maximal (Barr, Levy, Scheepers & Tily, 2013). Using R syntax, the model tested was invRT ~ Prime type * Similarity * Target Context + PrevRT + (Similarity | subject) + (Similarity | target), with 47 subjects and 24 targets. After removing RTs on error trials from both the current and previous trials, there were 4118 data observations.

The results of the model are summarized in Table 2.^1^ Critically, there was a significant triple interaction between Prime type, Similarity and Target context (t = 4.209, p < .001). To quantify the amount of evidence for the triple interaction, we calculated the Bayes factor using the BayesFactor package (Version 0.9.7, Morey & Rouder, 2013) available in R to compare two mixed effects models that differed in the inclusion of the interaction. Model 1was the mixed effects model described above, and Model 2 did not include the triple interaction. We then used the “compare” function with the default JZS prior to calculate the Bayes factor using Model 2 as the denominator. The Bayes factor was 403.2296 ±7.8 %. A Bayes factor of 1 means equal evidence for the contrasting hypotheses (they are equally plausible), and according to Jeffereys’ (1961) recommendation, odds greater than three are considered “some evidence”, odds greater than ten “strong evidence”, and odds greater than 30 “very strong evidence.” Thus, the Bayes factor analysis indicated “very strong evidence” for the triple interaction, i.e., the hypothesis that the size of Similarity effect was modulated differently by Target context for the digit and letter primes. From Table 1, it appears that this pattern results from the presence of Similarity effects in all conditions, except the Letter primes in the Overlapping Target Context. We confirmed this observation by first conducting separate analyses of the Letter primes and Digit primes, using the model invRT ~ Similarity * Target Context + PrevRT + (Similarity | subject) + (Similarity | target), which confirmed the presence of a Similarity by Context interaction for Letter primes (t = 3.267, p < .01), but not for Digit primes (t = -1.64, p = .107). As the predictions from the activation-based and Bayesian accounts related to the simple effects of similarity for the four Prime Type by Context conditions, we further fit the reaction times from each cell to a model of the form *invRT~Similarity + PrevRT + (Similarity | Subject) + (Similarity | target)*. The results are consistent with the Bayesian account, but not the activation-based account. For digit primes, both the Distinct Target Context and Overlapping Target Context produced significant Similarity effects (t = −7.753, p < .001 and t = −6.858, p < .001 respectively). For Letter primes, the Distinct Target Context produced a significant Similarity effect (t = −3.56, p < .01) while the Overlapping Target Context did not (t < 1, p = .768.).

**Table 2:** The model’s estimate, standard error (Std. Error), degrees of freedom (df), t-value and p-values of fixed effects (inverse reaction times (RTs) were multiplied by −1 to give the parameter estimates the same interpretation as in raw RTs, and by 1000 to reduce the number of decimal places)

	Estimate	Std Error	df^a^	t-value	p-value
Intercept	−2.559	0.044	128	−58.49	<0.001
Prime	0.166	0.129	3979	12.875	<0.001
Similarity	−0.158	0.020	3	−7.745	<0.001
Target context	−0.037	0.067	4.4	−0.502	0.618
Previous trial RT	0.001	0.000	4081	11.95	<0.001
Prime × similarity	0.187	0.025	3984	7.26	<0.001
Prime × target context	0.117	0.025	3977	4.536	<0.001
Similarity × target context	0.032	0.033	4.4	.974	0.335
Prime × similarity × target context	0.216	0.051	3982	4.21	<0.001

^a^The degrees of freedom were estimated using Satterthwaite’s approximation as implemented in lmerTest (Kuznetsova, Brockhoff, & Christensen, 2013)

The error data were analyzed using a logit mixed model (Jaeger, 2008) with the same fixed factors (excluding Previous trial RT) included in the RT model, and subjects and item intercepts as crossed random factors. In this analysis, Similarity was significant (t = −2.944, p < .01), as was the interaction between Prime type and Similarity (t = 2.347, p < .05). Importantly, there were no significant interactions with Target Context to indicate a speed-accuracy tradeoff.

The “Different” trials were not analyzed, as the predictions concern only the “Same” trials.

### Discussion

The present study used a same-different letter string match task and showed that it is possible to obtain an effect of visual similarity with letter primes, depending on context. When the “Different” targets were made up of letters from the primes (the Overlapping Target Context), visually similar letter primes (e.g., HRL-ABI) produced no more priming than visually dissimilar letter primes (e.g., DWG-ABI), consistent with the lexical decision task (Kinoshita et al., 2014). In contrast, when the “Different” targets consisted of letters that were distinct from those used in the letter primes and referents (The Distinct Target Context), the visually similar letter primes produced more priming than the visually dissimilar letter primes.

The Bayesian account predicted exactly this pattern on the assumption that letter recognition is a dynamic process of accumulating evidence for a task-driven decision. When visually similar letters (e.g., A and H) need to be distinguished as in the Overlapping target context, the form of the letter H does not contribute evidence for the presence of the visually similar letter A, because it serves as evidence for the competing hypothesis that the input is the letter H. In contrast, in the Distinct target context, the letter H need not be distinguished from the letter A, and hence its visual similarity to the letter A can contribute towards the evidence that the input is the letter A. This modulation of similarity effects by Target context was specific to the letter primes: Digit primes showed a robust effect of similarity, irrespective of target context, since digits never appeared in the referents or targets in either target context – that is, it was never necessary to consider a hypothesis that the input was a digit.

One alternative explanation of the observed results is in terms of response bias. That is, in the Overlapping context in which the letters HRL appeared as “Different” targets, the prime HRL caused a bias towards responding “Different” and this offset the priming benefit due to its similarity to the referent/target ABI. As noted in the Introduction, there is extensive evidence against the response bias explanation for absence of priming (see e.g., Norris & Kinoshita, 2010; Kinoshita & Norris, 2010, 2011). Here also, the finding of robust similarity effect for digit primes in the Overlapping context condition rules out this explanation. The “similar digit” prime (e.g., 481) is visually more similar to the “Different” target HRL than the “dissimilar digit” prime 673. Thus, if a bias towards responding “Different” to HRL offset the similarity-based priming effect for the letter primes, this should have similarly abolished the similarity effect for digit primes. Clearly, response bias cannot explain the dissociation between digit and letter primes.

The present results shed a light on why in previous lexical decision studies visual similarity effects have been observed readily with digit primes (“leet primes,” e.g., Perea et al., 2008), but not with letter primes (e.g., Kinoshita et al., 2014). In the lexical decision task, the targets are always letter strings, and letters must be distinguished from other letters. Digits never appear as targets in a lexical decision task, hence it is not necessarily to distinguish a digit from a visually similar letter. In other words, the task context of a lexical decision task is just like the Overlapping target context in the present experiment. We have noted earlier that it is possible to explain the dissociation between the digit and letter primes in that experiment by positing structural differences between digit and letter representations (e.g., by positing stronger letter-to-letter lateral inhibitory connections than letter-to-digit connections), but, the present context-dependent similarity effects are less readily explained by such structural differences. In the interests of parsimony, we suggest that the dissociation between digit and letter primes in the visual similarity effect is explained better in terms of task-dependent perceptual decision which guides the nature of information that serves as evidence for the decision.

The present results also have implications for the debate about whether letters are “special” visual objects. This debate has taken place in many contexts, including whether the brain area dubbed the “visual word form area” is selectively responsive to letters (e.g., Dehaene & Cohen, 2011; Price & Devlin, 2003), whether “pure alexia” patients have a selective deficit in recognition of letters (e.g., McCloskey & Schubert, 2014), and whether coding of letter position within a string is more or less precise than other visual objects (e.g., Duñabeitia, Dimitropoulou, Grainger, Hernández, & Carreiras, 2012). In this debate, the observation of dissociations between letters and other visual objects provide the main evidence for the view that letters are special. The present finding of a context-dependent dissociation even for letters suggests that this debate would benefit from considering the demands of the task contexts that produce the dissociation between letters and other visual objects.

## Electronic supplementary material

ESM 1 (DOCX 33 kb)

## Footnotes

1 P-values were estimated using the lmerTest package; Kuznetsova, Brockhoff, & Christensen, 2013, version 2.0-11
