# Supplementary material for: Context-dependent similarity effects in letter recognition
Source: Psychon Bull Rev. 2015 Apr 9;22(5):1458–64. doi: 10.3758/s13423-015-0826-3 (PMC4577525; doi:10.3758/s13423-015-0826-3)
Supplement: Supplementary file 1 — (DOCX 33 kb) [file 13423_2015_826_MOESM1_ESM.docx]

Supplementary material

Appendix

Critical stimuli used

Targets were used as Referent and “Same” targets. Identity = identity prime, SimL = Similar letter prime, DisL = Dissimilar letter prime, DimD = Similar digit prime, DisD = Dissimilar digit prime, Diff Target Distinct = “Different” targets used in the Distinct context, Diff Target Overlapping = “Different” targets used in the Overlapping context

Referents and primes were presented in 10 point Courier New font; the targets were presented in 12 point Courier New font

| Target | Identity | SimL | DisL | SimD | DisD | Diff Target  Distinct | Diff  Target  Overlapping |
| --- | --- | --- | --- | --- | --- | --- | --- |
| ABI | ABI | HRL | DWG | 481 | 673 | CZE | HRL |
| ABS | ABS | HRE | DWM | 485 | 679 | CZY | HRE |
| AIB | AIB | HLR | DGW | 418 | 637 | CEZ | HLR |
| AIS | AIS | HLE | DGM | 415 | 639 | CEY | HLE |
| ASB | ASB | HER | DMW | 458 | 697 | CYZ | HER |
| ASI | ASI | HEL | DMG | 451 | 693 | CYE | HEL |
| BAI | BAI | RHL | WDG | 841 | 763 | ZCE | RHL |
| BAS | BAS | RHE | WDM | 845 | 769 | ZCY | RHE |
| BIA | BIA | RLH | WGD | 814 | 736 | ZEC | RLH |
| BIS | BIS | RLE | WGM | 815 | 739 | ZEY | RLE |
| BSA | BSA | REH | WMD | 854 | 796 | ZYC | REH |
| BSI | BSI | REL | WMG | 851 | 793 | ZYE | REL |
| IAB | IAB | LHR | GDW | 148 | 367 | ECZ | LHR |
| IAS | IAS | LHE | GDM | 145 | 369 | ECY | LHE |
| IBA | IBA | LRH | GWD | 184 | 376 | EZC | LRH |
| IBS | IBS | LRE | GWM | 185 | 379 | EZY | LRE |
| ISA | ISA | LEH | GMD | 154 | 396 | EYC | LEH |
| ISB | ISB | LER | GMW | 158 | 397 | EYZ | LER |
| SAB | SAB | EHR | MDW | 548 | 967 | YCZ | EHR |
| SAI | SAI | EHL | MDG | 541 | 963 | YCE | EHL |
| SBA | SBA | ERH | MWD | 584 | 976 | YZC | ERH |
| SBI | SBI | ERL | MWG | 581 | 973 | YZE | ERL |
| SIA | SIA | ELH | MGD | 514 | 936 | YEC | ELH |
| SIB | SIB | ELR | MGW | 518 | 937 | YEZ | ELR |
